# Hesperetin mitigates sorafenib-induced cardiotoxicity in mice through inhibition of the TLR4/NLRP3 signaling pathway

**DOI:** 10.1371/journal.pone.0271631

**Published:** 2022-08-09

**Authors:** Dalia Zaafar, Heba M. A. Khalil, Rabab Ahmed Rasheed, Rania Farag A. Eltelbany, Sawsan A. Zaitone

**Affiliations:** 1 Department of Pharmacology and Toxicology, Faculty of Pharmacy, Modern University for Information and Technology, Cairo, Egypt; 2 Department of Veterinary Hygiene and Management, Faculty of Vet. Medicine, Cairo University, Giza, Egypt; 3 Department of Histology and Cell Biology, Faculty of Medicine, King Salman International University, South Sinai, Egypt; 4 Department of Biochemistry, Faculty of Pharmacy, Modern University for Information and Technology, Cairo, Egypt; 5 Department of Pharmacology & Toxicology, Faculty of Pharmacy, Suez Canal University, Ismailia, Egypt; Virginia Commonwealth University, UNITED STATES

## Abstract

Sorafenib is an oral multi-kinase receptor inhibitor that targets various signaling pathways. It is used as the first line of treatment in advanced hepatocellular and renal cell carcinomas. Sorafenib was reported to induce cardiotoxicity due to myocyte necrosis. Hesperetin is a naturally occurring flavonoid with antioxidant and anti-inflammatory capabilities. This study investigated the putative protective effect of hesperetin against sorafenib-induced cardiotoxicity in mice through downregulation of NLRP3/TLR4 signaling and inhibition of apoptosis. Twenty-four male Swiss mice were distributed into four groups: untreated control, hesperetin (50 mg/kg/day, orally), sorafenib (100 mg/kg/day, orally), and combination (Hesperetin+Sorafenib). After a three-week treatment period, various biochemical parameters in cardiac tissues were assessed. TNF-α, IL-1β, and IL-6 levels were measured. Moreover, TLR4 and NLRP3 expressions were evaluated using Western blot analysis. Histopathological examination and immunohistochemical assessment of apoptotic activity were done. Compared with the sorafenib group, the combination group exhibited reduced TNF-α, IL-1β, IL-6 levels and lower NLRP3/TLR4 expressions. Histologically, the combination group showed improved myocardial histology and a marked decrease in collagen deposition. Immunohistochemical examination showed decreased caspase-3 and increased Bcl-2 expression. Before recommending hesperetin as an adjuvant, clinical studies are warranted for mitigating sorafenib cardiotoxicity.

## Introduction

In recent years, advances in antineoplastic therapy procedures, particularly developing a series of innovative anticancer medications, have considerably improved cancer prognosis. Despite the potential of tumor elimination and more extended longevity, anticancer drug-induced adverse effects, notably cardiotoxicity, including heart failure, cardiomyopathy, arrhythmias, thromboembolic events, acute coronary syndrome, and myocardial infarction, are a major health hazard [1, 2].

Sorafenib is an oral multi-kinase inhibitor that inhibits tyrosine kinase receptors to target multiple pathways involved in angiogenesis and tumor growth. Sorafenib is suggested as a first-line treatment for advanced hepatocellular and renal cell carcinomas [2]. Cardiomyocyte necrosis is the main pathogenic mechanism of sorafenib cardiotoxicity.

Sorafenib’s pharmacokinetics show high inter-individual variability, with varying absorption (time to maximum concentration ranging from 2–12.5 h) and secondary peaks, as well as a long elimination half-life (20–39 h) in cancer patients [3]. CYP3A4 and UGT1A9 convert sorafenib into seven different metabolites by oxidative metabolism and glucuronidation respectively. Sorafenib N-oxide, a product of CYP3A4-mediated metabolism, is the main circulating metabolite in blood plasma [4, 5]. Sorafenib pharmacokinetic variability could be attributed to low hepatic extraction ratio and high protein-binding ability (>99.5%).

Although it has been observed that sorafenib-related cardiac adverse effects are frequently transitory and medication can be continued [5], some studies suggest the addition of supplements to sorafenib therapy to reduce its negative impact on the cardiac muscles [6].

Notably, the use of combination therapies such as traditional herbal medicines or natural compounds to improve the clinical outcomes and reduce adverse events of sorafenib therapy has emerged as a novel therapeutic approach [7].

Hesperetin is a naturally occurring flavonoid molecule generated predominantly from the hydrolysis of hesperidin (4′-methoxy-3′,5,7-trihydroxyflavanone). It is found in citrus fruits, consumed due to its high content of vitamins and flavonoids with variant biomedical potentials [8]. According to Liu et al., hesperetin had a cardioprotective effect in an animal model of myocardial ischemia partly mediated by antioxidant and anti-inflammatory capabilities. It could penetrate phospholipid bilayers and scavenge free oxygen radicals [1]. Moreover, several research reported the antiviral, anticancer, and neuroprotective properties of hesperetin [9–12]. Based on the foregoing, we anticipated that hesperetin has a promising action in mitigating the sorafenib-induced cardiotoxicity.

Reducing the release of different inflammatory cytokines such as interleukin 1-β (IL-1β), interleukin-6 (IL-6), interleukin 18 (IL-18), and tumor necrosis factor-alpha (TNF-α) can guard against cardiac tissue inflammation [13, 14]. In addition, the Toll-like receptor 4/ Nuclear factor-kappa B (TLR4/NF-kB) signaling pathway is implicated in generating inflammatory responses and the pathophysiology of myocardial ischemia injury via modulating pro-inflammatory cytokine production [13]. TLR4 activation causes NF-kB to rise, which controls the expression of the pro-inflammatory cytokines [14]. Furthermore, after myocardial infarction, nucleotide-binding domain leucine-rich repeat (NLR) and pyrin domain-containing receptor 3 (NLRP3) inducts into the heart and amplifies the inflammatory response [15]. The NLRP3 inflammasome is the most characterized studied inflammasome and is made up of a nucleoside binding oligomeric domain-like receptor (NLRP3), a caspase recruitment domain (ASC), and caspase-1 [16]. Significantly the activation of the NLRP3 pathway can be induced by TLR4 and inflammatory cytokines receptors like TNF-α. Upon NLRP3 activation, it induces the secretion of inflammatory cytokines like IL-1β [17]. Consequently, NLRP3 activation occurs in several cardiovascular diseases [18]. Furthermore, caspase-1 translocation and activation were attained after NLRP3 protein polymerization and coupling to the ASC adaptor. In addition, mature versions of pro-inflammatory cytokines are released by activated caspase-1. As a result, active IL-1 and IL-18 were released into the extracellular space due to activated caspase-1 cleaving of IL-1 and IL-18 precursors, which recruit inflammatory cells and prolong the inflammatory response [6]. Negative modulation of the TLR4/NLRP3 signaling pathway, on the other hand, may have cardioprotective advantages by lowering the inflammatory response [13].

In the current study, we investigated the role of hesperetin in mitigating sorafenib-induced cardiotoxicity in mice and tested whether downregulating NLRP3/TLR4 signaling and antiapoptotic activity play a role in this putative cardioprotective effect.

## Animals and methods

### Animal housing and ethical statement

Male Swiss mice (25–27 g) were provided from the National Cancer Institute (Cairo, Egypt) and were acclimatized before starting the experiments for one week. They were kept under standard laboratory conditions (temperature, 25°C; humidity, 50%, and normal light/dark cycle). Water and a well-balanced commercial diet were freely available *ad libitum*. The experimental procedures were performed in accordance with the NIH Guidelines for the Care and Use of Laboratory Animals and approved by the Institutional Animal Care and Use Committee (Vet-IACUC) of Cairo University.

### Medications

Sorafenib was obtained in tablet form (Nexavar, Bayer AG, Berlin, Germany). Each tablet contained 200 mg of sorafenib and was ground with a mortar and suspended in 2% CMC solution. Hesperetin was supplied from Sigma-Aldrich (St. Louis, MO, USA), suspended in ice-cold 2% carboxymethyl cellulose (CMC) solution, and divided into aliquots containing working solutions that were immediately frozen at −20°C. Each aliquot was allowed to melt just prior to use.

### Experimental study

Twenty-four mice were randomly distributed in four groups (n = 6) as follows: Group I (Vehicle control): mice were orally administered the vehicle of sorafenib (2 ml/kg, 2% CMC solution), group II (hesperetin group): mice were administered hesperetin (50 mg/kg/day), group III (Sorafenib group): mice were administered sorafenib (100 mg/kg daily), and group IV (combination group): mice were administered sorafenib (100 mg/kg/day) and hesperetin (50 mg/kg/day). In general, drug treatments were given by oral gavage and continued for 21 days. More specifically, sorafenib was administered at 8 AM, whereas hesperetin was given at 3 PM on each day; the control group received equivalent volumes of 2% CMC solution at the same time points parallel to the drug regimens.

### Justification of sorafenib dose

The dose of sorafenib was in accordance with previously reported doses in mice [16, 21].

### Euthanasia and tissue sampling

Blood was obtained from the orbital canthus 24 h after the last drug administration, and serum samples were separated. The mice were then euthanized using ketamine (Dopalen^®^ 300 mg/kg) and xylazine (Anasedan^®^ 30 mg/kg, i.p.) anesthesia and cervical dislocation. A thoracotomy was performed to dissect the hearts, and each heart was divided longitudinally into two halves. The first half was fixed in formol saline (10%), whereas the second half was immediately placed at −80°C for biochemical analysis.

### Biochemical analysis

#### Assessment of TNF-α, IL-1β, and IL-6 levels in serum

Frozen cardiac tissue samples were homogenized in a 10% phosphate-buffered saline (PBS) solution using a Teflon homogenizer, and the homogenates were centrifuged for 10 min at 2000 × g. The clear supernatants were frozen in Eppendorf tubes until further use in ELISA. According to the instructions of Abbexa (Cambridge, UK), TNF-α levels were determined using a sandwich enzyme-linked immune-sorbent assay (Catalog no. abx050220). The IL-1β and IL-6 ELISA kits were used according to the instructions from Cloud-Clone Corp, USA (Catalog numbers SEA563Ra and SEA079Ra, respectively). The concentration of each marker was estimated after measuring optical density with an automated ELISA reader at 450 nm.

#### Western blot analysis

To produce heart tissue lysates for Western blot analysis, a lysis buffer containing 50 mM Tris-HCl, 150 mM NaCl, 1 mM EDTA, 1 mM EGTA, pH 7.8, and 1 percent Triton X100 supplemented with sodium orthovanadate, sodium pyrophosphate, protease inhibitor cocktail, and PMSF was utilized. TLR4 (Santa Cruz Biotechnology, #sc-10741), NLRP3 (Abcam, USA, #ab263899), Caspase-3 (Santa Cruz Biotechnology, #sc-56053), BcL2 (Santa Cruz Biotechnology, #sc-7382), and Transforming growth factor β (TGFβ) (Santa Cruz Biotechnology, # sc-130348) antibodies were used. The intensity of the protein bands was standardized to β-actin, evaluated with ImageJ software (NIH, USA), and the data was reported as a percentage compared with controls.

### Histopathological and immunohistochemical examination

Specimens from the left ventricles of mice from various research groups were fixed in formol saline (10%), processed, and paraffin-embedded to obtain blocks. Based on Suvarna and colleagues, 6-mm-thick sections were cut and stained with hematoxylin and eosin (H&E) for interpreting histopathological alterations and Masson’s trichrome stains for detecting collagen [19]. In addition, immunohistochemical staining was performed after antigen retrieval. Briefly, the sections were boiled in 10 mM buffer (AP9003) for ten min at pH 6 and incubated for one hour with primary antibodies for caspase (rabbit polyclonal antibody, ab13847) and Bcl-2 (rabbit polyclonal antibody, ab196495). Both exhibited cytoplasmic reactions and were purchased from Abcam, MA, USA. Immunostaining was done using an Ultra vision detection system (TP-015-HD), and finally, counterstaining with Mayer’s hematoxylin (catalog number TA- 060-MH) was performed. Negative controls were created by removing the main antibody. The citrate buffer, Ultra vision detecting system, and Mayer’s hematoxylin was obtained from Lab Vision Thermo Scientific, Fremont, California, USA. Morphometric studies were done to measure the mean area percentage of collagen fibers in Masson’s trichrome-stained sections (X 200), Bcl-2, and caspase-3 expression in immune-stained sections (X 400) by randomly choosing ten non-intersecting fields from different slides of the study groups. Measurements were performed in the Department of Pathology, National Research Center, Cairo using NIH image J software ver. 1.50i, USA, and snapped by Leica Qwin-500 LTD-software image analysis computer system Ltd. (Cambridge, England).

### Statistical analysis

The data were presented as means ± standard deviation. Statistical analysis was assessed using version 24 of the SPSS program (software; SPSS Inc., Armonk, NY, USA). Comparisons between groups were made by applying one-way analysis of variance (ANOVA) followed by Tukey’s post hoc test. The statistical significance level was set at P ≤ 0.05.

## Results

### Effect of sorafenib and/or hesperetin on serum levels of the inflammatory markers

**Fig 1:**
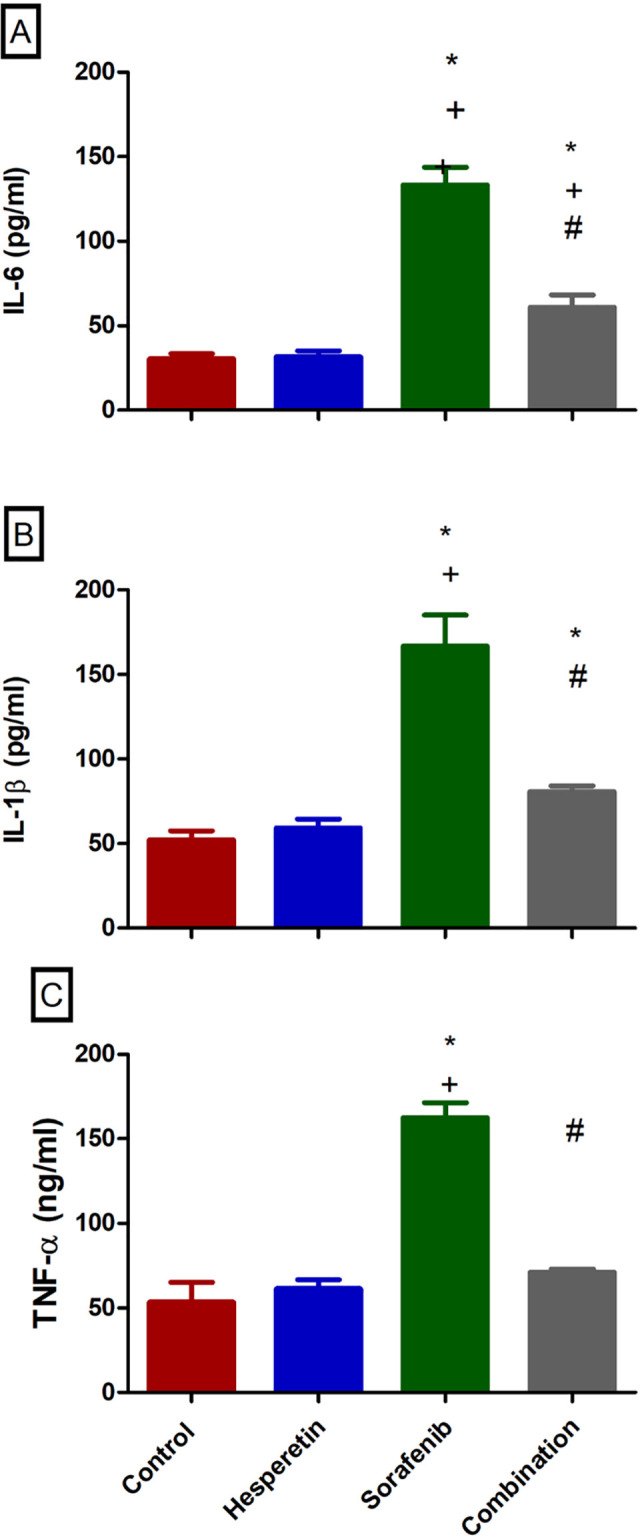
Effect of sorafenib alone or added to hesperetin on the serum levels of different inflammatory biomarkers (a) Interleukin 6 (IL-6), (b) Interleukin 1β (Il-1β), and (c) Tumor necrosis factor (TNF-α) in different mice groups. Data are expressed as mean ± SD, one-way ANOVA followed by post hoc Tukey’s test. * Significant from the control group, + Significant from the hesperetin group, # Significant from the sorafenib group, *p*<0.05.

The current investigation found that when hesperetin was added to sorafenib, IL-6 levels were considerably lower than the sorafenib-treated group (60.9±7.4) vs. (133.3±10.5), respectively. As shown in (Fig 1), serum levels of IL-1β and TNF-α were reduced in the combination group (80.7±3.4) and (71.1±1.9) as compared to the markedly raised levels in the sorafenib-treated group (166.7±18.3) and (162.5±8.9) respectively.

### Effect of sorafenib and/or hesperetin on the cardiac expression of NLRP3 inflammasome and TLR4

**Fig 2:**
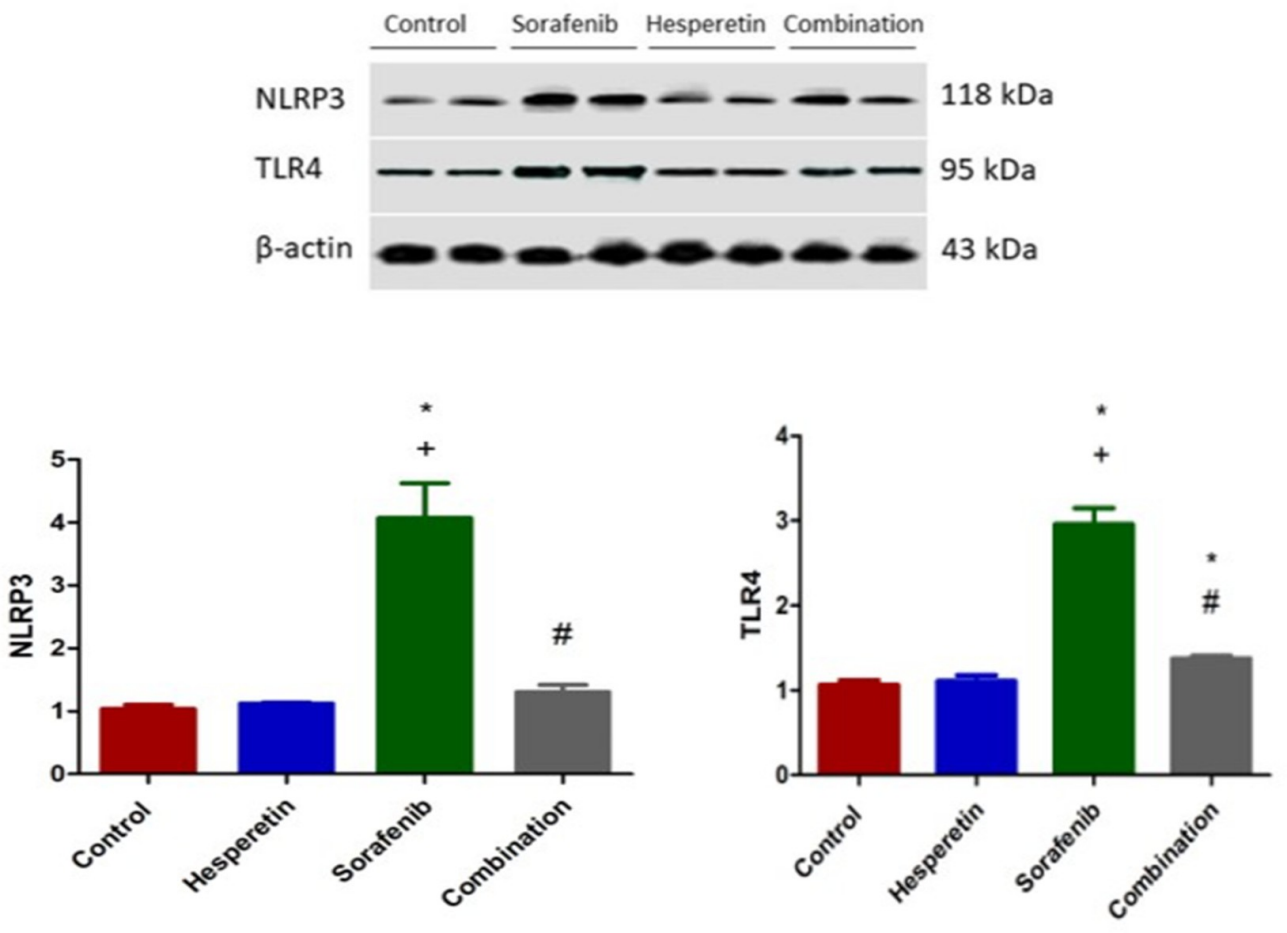
Effect of sorafenib alone or added to hesperetin on the expression of NLRP3 inflammasome and TLR4 via western blotting in different groups. Data are expressed as mean ± SD, one-way ANOVA followed by post hoc Tukey’s test. * Significant from the control group, + Significant from the hesperetin group, # Significant from the sorafenib group, *p*<0.05.

Our recent findings indicated that the sorafenib-treated group showed an upregulated level of cardiac NLRP3 protein. Co-administration of hesperetin with sorafenib (1.3±0.12) significantly reduced these protein levels compared with the other groups (1.037±0.063), (1.12±0.018), (4.1±0.55) respectively as shown in (Fig 2). The administration of sorafenib alone resulted in a significant increase in cardiac TLR4 protein (3±0.2). In contrast, the group treated with the combination of sorafenib and hesperetin exhibited significantly lower levels of TLR4 protein (1.4±0.03) (Fig 2).

### Effect of sorafenib and/or hesperetin on the cardiac expression of Caspase-3, BcL2 and TGFβ

**Fig 3:**
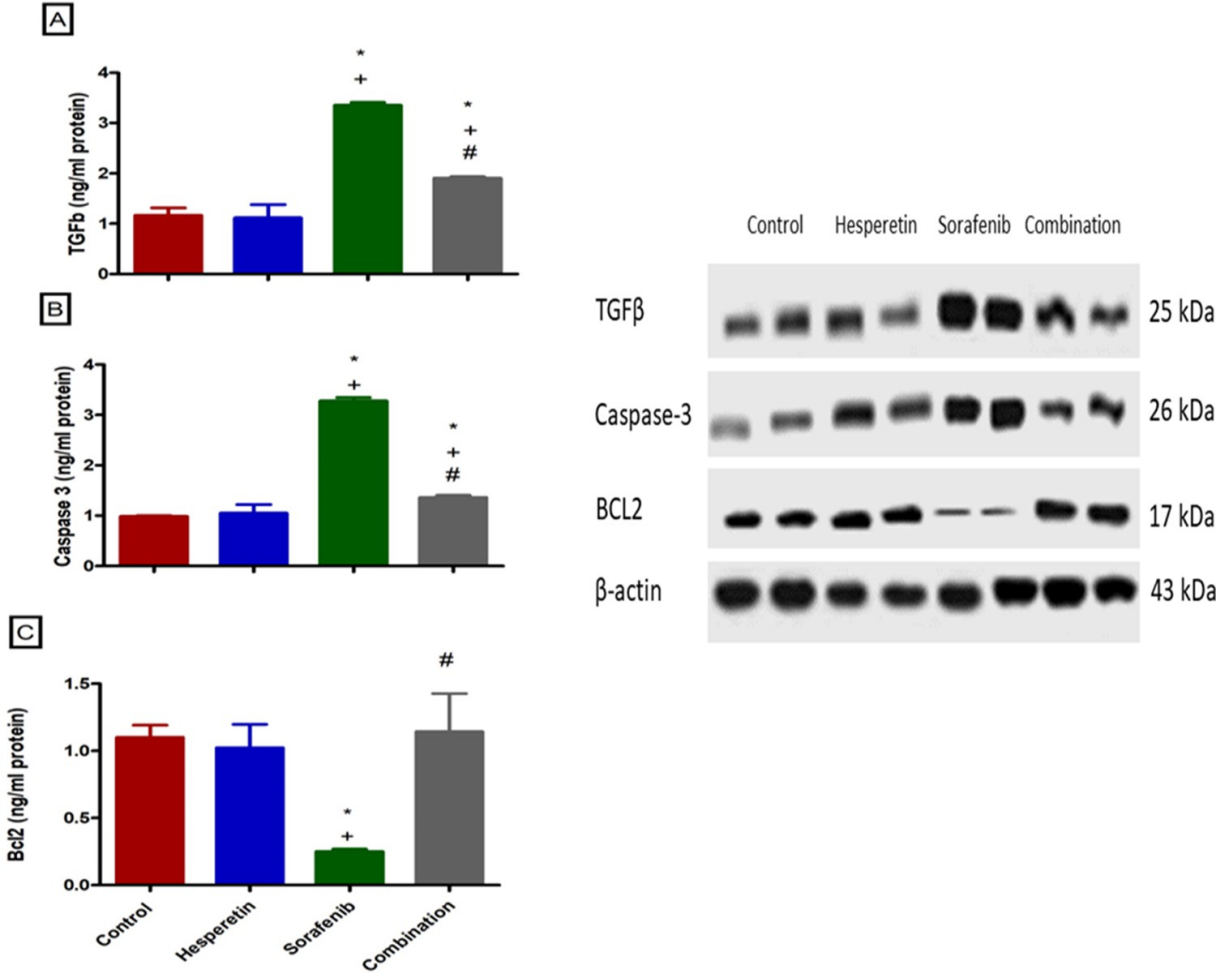
Effect of sorafenib alone or added to hesperitin on the expression of (a) Caspase-3 (b) BcL2 (c) TGF-b via western blotting in different groups. Data are expressed as mean ± SD, one-way ANOVA followed by post hoc Tukey’s test. * Significant from the control group, + Significant from the hesperitin group, # Significant from the sorafenib group, *p*<0.05.

The current findings highlighted the upregulated level of the growth factor TGFβ in the mice treated with sorafenib alone (3.35±0.06) while when hesperetin was added it could successfully down regulate TGFβ levels significantly (1.9±0.03) (Fig 3A).

Additionally, a significant upregulation in the level of the apoptotic biomarker (Caspase-3) was demonstrated in the cardiac tissue of the sorafenib treated mice (3.28±0.06) compared with the normal, hesperetin and combination treated rats (0.99±0.01), (1.05±1.2) and (1.4±0.04) respectively. On the other hand, the BcL2 biomarker was significantly inhibited in the sorafenib treated mice (0.25±0.02) which was then normalized by adding hesperetin to sorafenib (1.1±0.03) (Fig 3B and 3C).

### Effect of sorafenib and/or hesperetin on the histopathological picture of cardiac tissues

**Fig 4:**
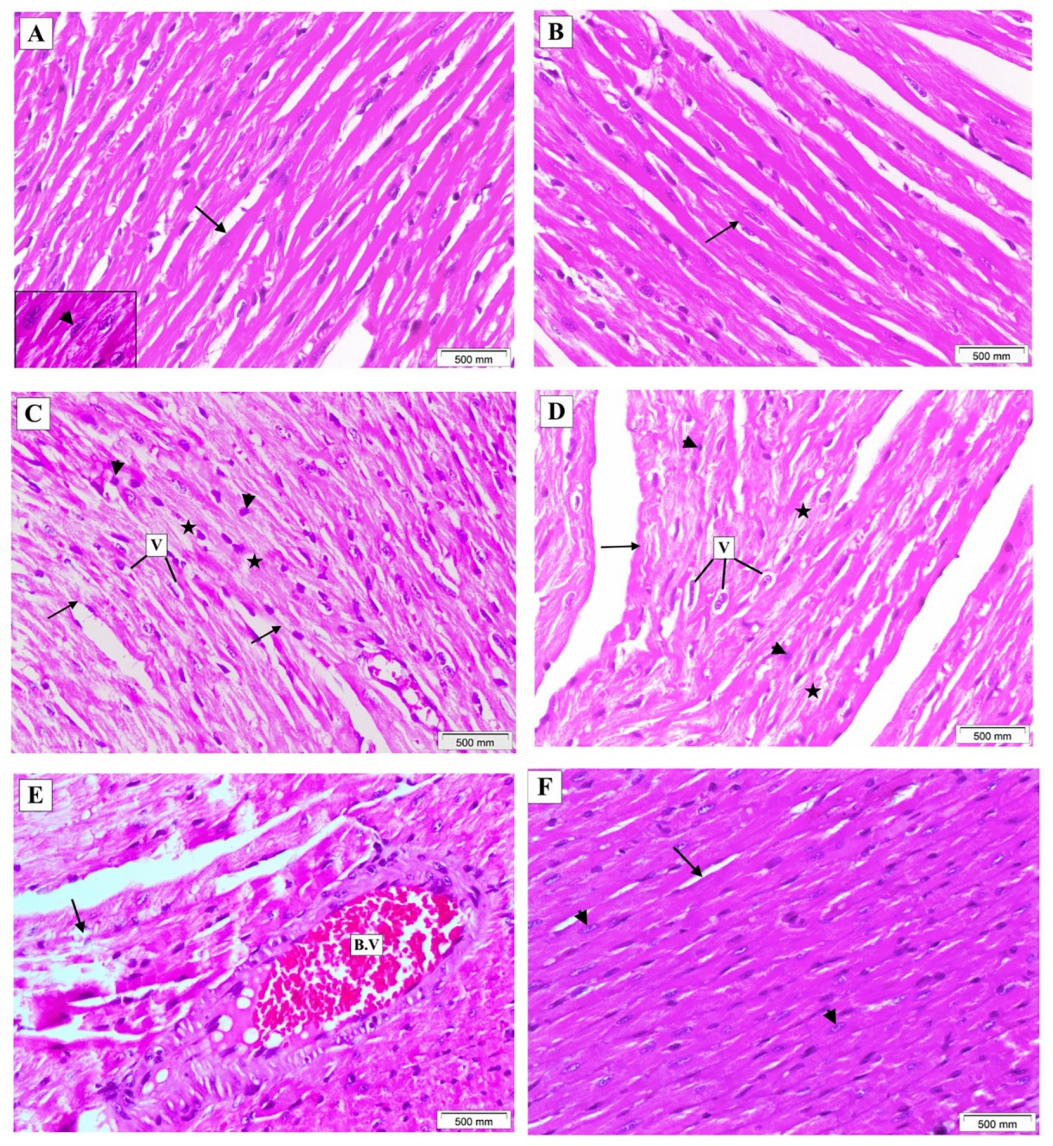
Histopathological changes induced by sorafenib in cardiac muscles in different groups; (A and B) Sections from the control and hesperetin groups, respectively, showing normal cardiac architecture with cylindrical branching cardiomyocytes (arrow). (inset X 400): oval centrally located nucleus (arrowhead). (C-E) Sections from the sorafenib-treated group showing degenerated hypertrophied cardiomyocytes with disrupted myofibrils (arrows). The sarcoplasm shows marked perinuclear vacuolization (V) and areas of focal lysis (stars). The nuclei are fading in some myocytes and pyknotic in others (arrowheads). Blood vessels are markedly engorged with thickened hyalinized walls (B.V). (F) Section from the combination group showing an almost normal histologic pattern of cardiac tissue with mildly hypertrophied cardiomyocytes (arrow) with normally looking nuclei (arrowheads). (H&E X 200).

**Fig 5:**
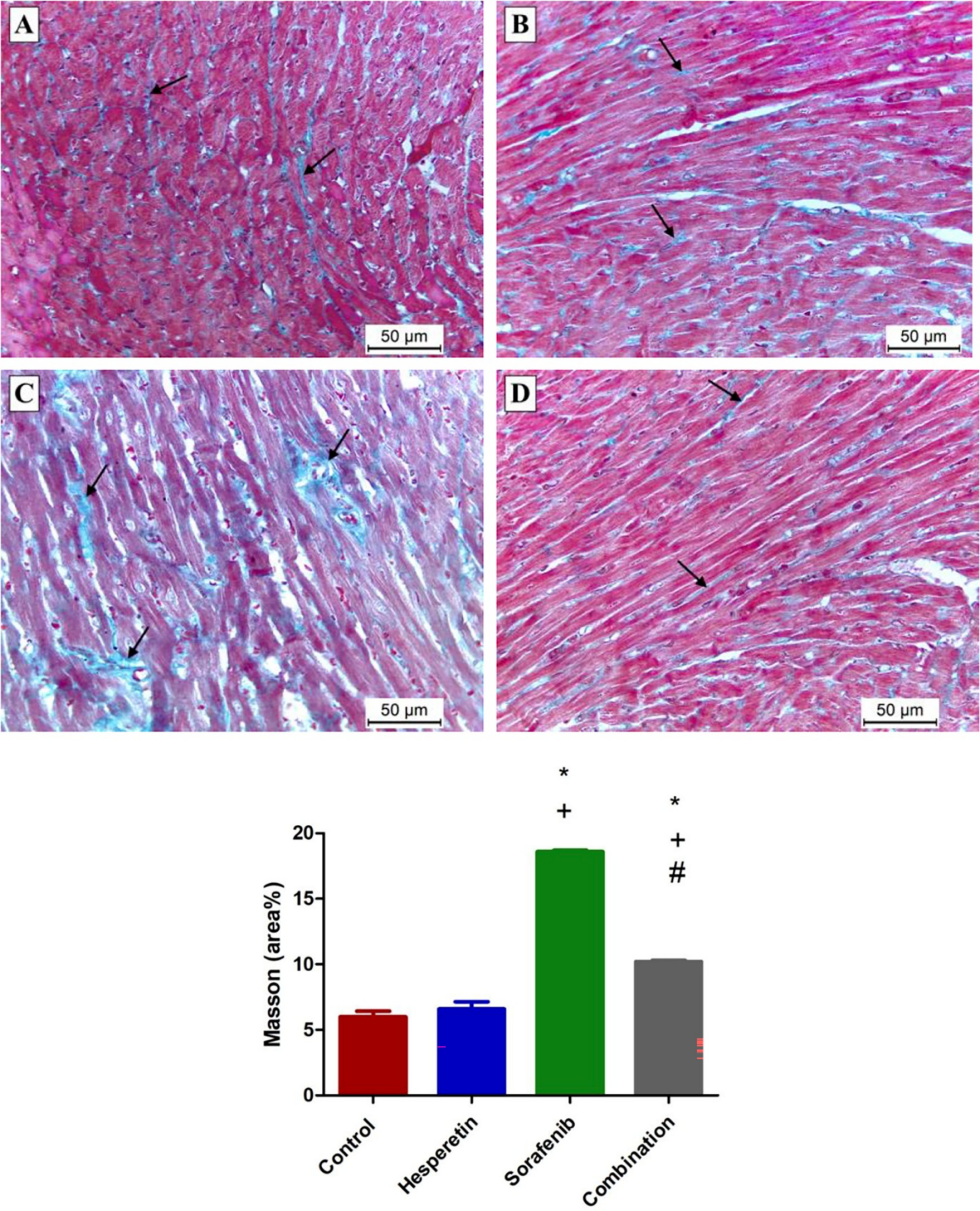
Photomicrographs representing Masson’s trichrome stained sections: (A and B) Minimal collagen fibers in between cardiomyocytes in the control and hesperetin groups, respectively (arrows). (C) Marked increase in interstitial collagen fibers in sorafenib group (arrows). (D) Little collagen fibers in the combination group (arrows). Data are expressed as mean ± SD, one-way ANOVA followed by post hoc Tukey’s test. * Significant from the control group, + Significant from the hesperetin group, # Significant from the sorafenib group, *p*<0.05. [Masson’s trichrome stained X 200].

Hematoxylin and eosin-stained sections from control and hesperetin groups showed normal cardiac architecture with cylindrical branching cardiomyocytes and an oval centrally located nucleus. The sorafenib-treated group showed degenerated hypertrophied cardiomyocytes with disrupted myofibrils, marked sarcoplasmic perinuclear vacuolization, and areas of focal lysis. The nuclei were faded in some myocytes and pyknotic in others. Blood vessels showed marked congestion with thickened hyalinized walls. The combination group had a nearly normal histologic pattern of cardiac tissue with mildly hypertrophied cardiomyocytes. The nuclei were normally looking like that of the control group, as shown in (Fig 4). Masson’s trichrome-stained sections showed minimal blue collagen fibers in between cardiomyocytes in both the control and hesperetin groups (6±0.4) and (6.6±0.5), respectively. The sorafenib-treated group exhibited a significant increase in interstitial collagen fibers deposition (18.6±0.1). In contrast, collagen deposition was significantly decreased in the combination group (10.2±0.1), as shown in (Fig 5).

### Effect of sorafenib and/or hesperetin on immunohistochemical staining for Bcl-2 and caspase-3 in cardiac section

**Fig 6:**
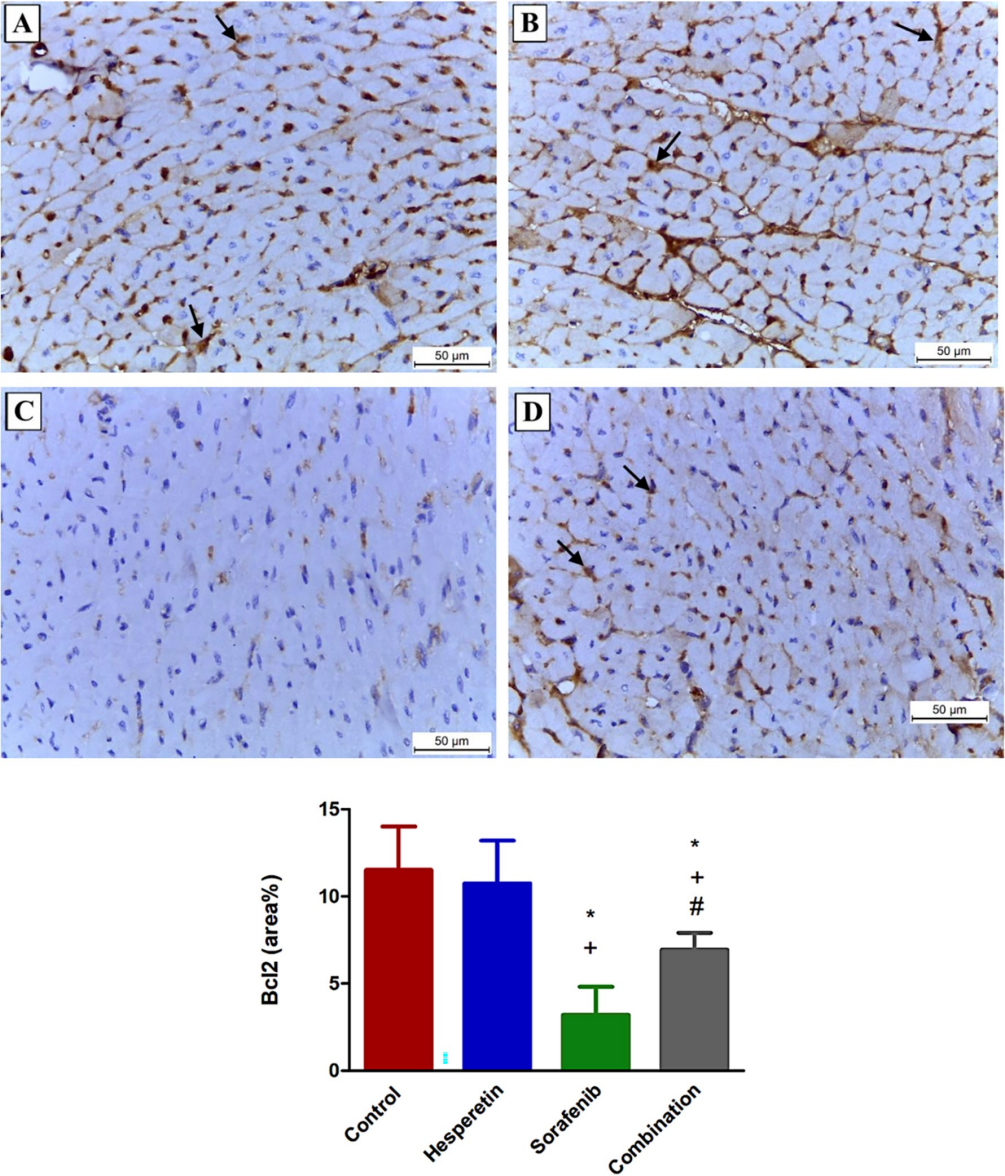
Photomicrographs representing Bcl-2 immunoreactivity in the sarcoplasm of cardiomyocytes of mice of different groups. (A and B) Sections from the control and hesperetin groups, respectively, showing positive sarcoplasmic Bcl-2 immunoreactivity (arrows). (C) Section from sorafenib group revealing negative cytoplasmic Bcl-2 immunoexpression. (D) Section from the combination group demonstrating positive Bcl-2 immunoexpression (arrows). Data are expressed as mean ± SD, one-way ANOVA followed by post hoc Tukey’s test. * Significant from the control group, + Significant from the hesperetin group, # Significant from the sorafenib group, *p*<0.05. (Bcl-2 immunostaining X 200).

**Fig 7:**
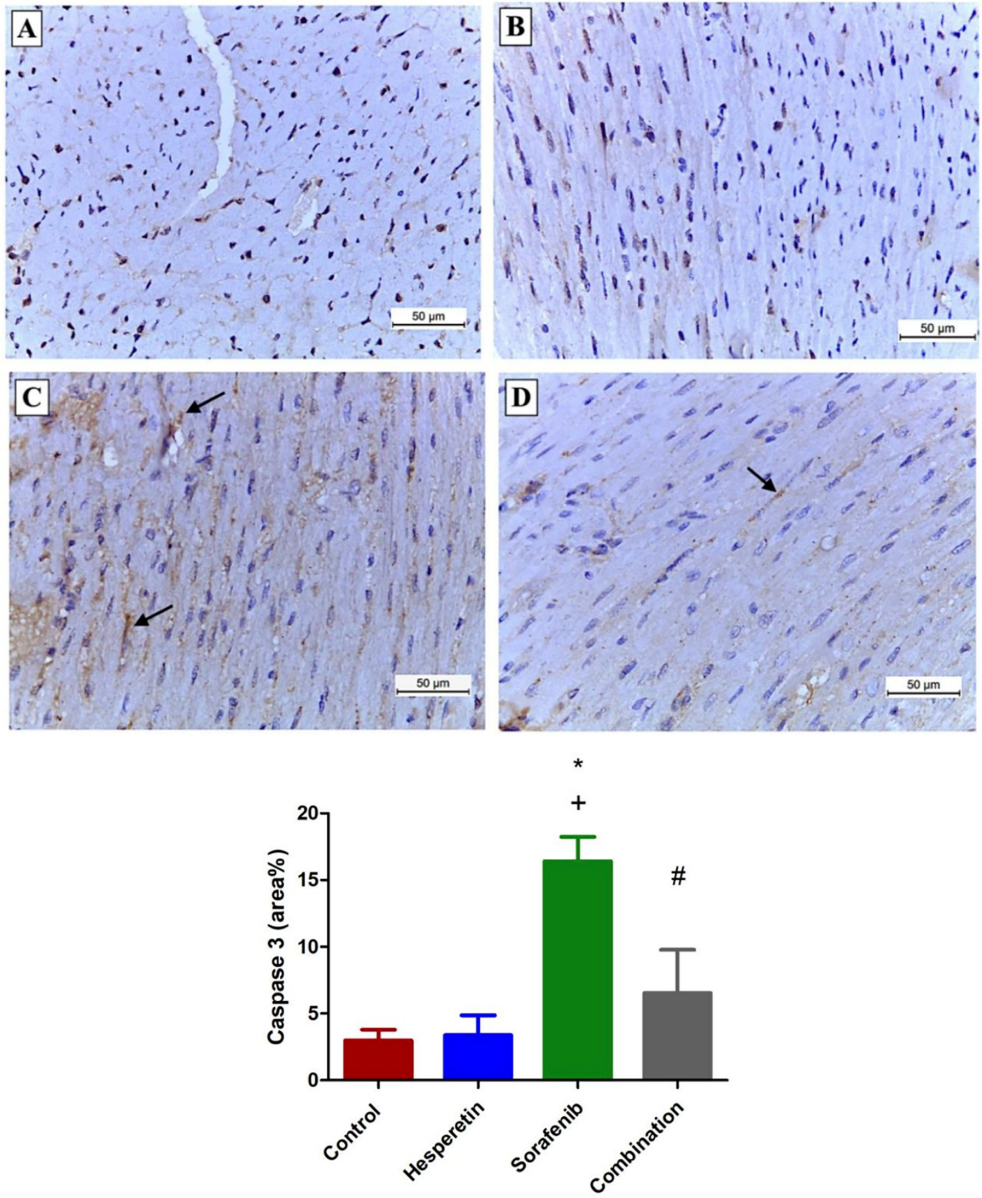
Photomicrographs representing caspase-3 immunoreactivity in the sarcoplasm of cardiomyocytes of mice of different groups. (A and B) Sections from the control and hesperetin groups, respectively, showing negative sarcoplasmic caspase-3 immunoreactivity. (C) Section from the sorafenib group displaying marked positive cytoplasmic caspase-3 immunoexpression (arrows). (D) Section from the combination group exhibiting mild positive caspase-3 immunoexpression (arrows). Data are expressed as mean ± SD, one-way ANOVA followed by post hoc Tukey’s test. * Significant from the control group, + Significant from the hesperetin group, # Significant from the sorafenib group, *p*<0.05. (Caspase-3 immunostaining X 200).

Both the control and hesperetin groups showed positive cytoplasmic immunoreactivity of Bcl-2 in many cardiomyocytes (11.5±2.5) and (10.8±2.5), respectively, whereas immunoreactivity was significantly decreased in the sorafenib-treated group (3.2±1.6). The combination group exhibited significant immunoreactivity for Bcl-2 in many fibers (7±1), as shown in (Fig 6). Both the control and hesperetin-treated groups demonstrated negative cytoplasmic immunoreactivity of caspase-3 (3±0.8) and (3.4±1.5), respectively, whereas, in the sorafenib-treated group, most cardiomyocytes exhibited significant positive expression (16.4±1.8). The combination group exhibited a significant reduction in cytoplasmic caspase-3 expression (6.5±3.2), as shown in (Fig 7).

## Discussion

In the present study, we evaluated sorafenib-induced cardiotoxicity in mice and the impact of administering hesperetin to mitigate this toxicity. The results highlight the biochemical and the histopathological changes in the hearts of mice receiving sorafenib. Biochemical investigations reported that sorafenib administration has resulted in increased TNF-α, IL-1, and IL-6 inflammatory cytokines, as well as upregulated levels of NLRP3, TLR4 and TGFβ proteins in cardiac tissues. Histopathological examination showed degenerated hypertrophied cardiomyocytes with disrupted myofibrils, sarcoplasmic perinuclear vacuolization, and areas of focal lysis. The nuclei were faded in some cardiomyocytes and pyknotic in others. The blood vessels showed marked congestion with thickened hyalinized walls. Masson’s trichrome staining showed fibrotic changes and collagen deposition in between the cardiomyocytes. Both western blotting and immunohistochemical examination showed a rise in caspase 3 expression, whereas Bcl-2, the anti-apoptotic protein, was reduced in the sorafenib-treated mice.

Sorafenib-induced cardiac damage has yet to be fully understood. However, Ma and colleagues provided direct evidence that sorafenib causes cardiotoxicity in rats by triggering excessive oxidative stress in cardiomyocytes, resulting in myocardial interstitial fibrosis, lytic necrosis, and marked cardiomyocyte hypertrophy [1]. Hence, finding agents that can counteract sorafenib’s adverse effects will help their reduction in the clinic.

In the same context, Abd-elgalil and colleagues reported that sorafenib administration induced histopathological cardiac alterations, including myocardial disarray, edema, and inflammatory cellular infiltrates [6]. Natural products, including hesperetin flavonoid, exhibit protective effects against chemotherapeutic agent-induced cardiotoxicity and markedly alleviate myocardial injury and apoptosis by reducing DNA damage and oxidative stress [20]; the results of our current investigation support those findings.

The body’s first line of defense is innate immunity which recognizes illness and initiates pathogen clearance and tissue restoration. The inflammasome is one of the most critical complexes involved in these processes. NLRP3, a multi-protein complex found in the inflammasome, binds pro-caspase-1 and subsequently cleaves pro-IL-1 and pro-IL-18 cytokine precursors into mature versions. When the inflammasome is activated, it promotes the inflammatory type of cell death [16].

Toll-like receptor 4 (TLR4), which is involved in pathogen recognition, and activation of immune and inflammatory responses, was investigated by Gugliandolo and colleagues for its role in regulating the host immune response and inflammatory pathways. They are thus critical components of the innate immune response, which are produced mainly by the inflammasome, a multi-subunit protein complex, along with IL-1 [21]. The production of pro-inflammatory cytokines like TNF-α and IL-1, which coordinate the activation of inflammatory cells, can activate multiple pathways when there is an overwhelming imbalance in the inflammatory response [22].

In macrophages, activation of the NLRP3 inflammasome involves two steps: priming and activation. Inflammatory stimuli, such as TLR4 agonists, commence the priming process by inducing NF-kB-mediated NLRP3 and pro-IL-1 production. The activation stage is triggered by the assembly of the NLRP3 inflammasome and the release of IL-1 and IL-18 by caspase-1 [23]. NF-kB signaling plays an important function in controlling the host response. When NF-kB is activated in response to pathogenic signals, it increases the transcription of several genes that code for proteins implicated in the immunological and inflammatory response [13].

In the present study, our findings provide evidence of the protective effect of hesperetin against sorafenib-induced cardiotoxicity. The results of western blot analysis and ELISA indicated a rise in IL-1β, IL-6, and TNF-α expression in the cardiac homogenates of sorafenib-treated mice, which was significantly downregulated with the co-administration of hesperetin with sorafenib.

A complex regulatory network links inflammatory cytokines and myocardial fibrosis, and the number of inflammatory factors is proportional to cardiac fibrosis severity. Several studies reported the cardioprotective effect of hesperetin through reducing the cardiac inflammatory response and protecting the damaged myocardium from exposure to overexpressed inflammatory cytokines, and removing the detrimental effect of inflammatory factors on causing cardiac fibrosis. Inflammatory cytokines and myocardial fibrosis are linked by a complex regulatory network, and the inflammatory factors level is proportional to the severity of cardiac fibrosis [18, 23]. Hesperetin anti-tissue injury activity has previously been linked to its suppression of NF-kB activation, which is a major regulator of the inflammatory response whose activation increases inflammation and causes the production of pro-inflammatory cytokines such as TNF-α, IL-1, and IL-6 [24].

Transforming growth factor β (TGFβ) is a proved pro fibrotic growth factor and cytokine. Several studies revealed the strong link between the increased TGFβ levels and the increased fibrosis especially in cardiac tissue [25–27]. Our study highlighted the significance elevation in TGFβ level in cardiac tissue of sorafenib treated mice reflecting the elevated levels of heart tissue fibrosis and consequently reduce the viability and contractility of cardiac tissue as showed in several studies [28, 29]. Successfully hesperetin down regulated the TGFβ levels when added to sorafenib showing a promising protecting effect on the heart tissue.

Overall, the cardiomyocytes’ pathology and inflammatory burden in mice following sorafenib treatment was significantly ameliorated by co-administration with hesperetin. We found that hesperetin mitigated sorafenib-induced cardiotoxicity histologically and biochemically by inhibiting the NLRP3/TLR4 signaling pathway and consequently down-regulating the inflammatory biomarkers regulated by this pathway, in addition to the down regulation of fibrotic and apoptotic biomarkers in cardiac tissue.

## Conclusion

To the best of our knowledge, the present study highlighted, for the first time, the potential protective effect of the natural flavonoid, hesperetin, against sorafenib-induced cardiotoxicity in mice and documented that this effect was -partly- mediated through downregulation of TLR4/NLRP3 signaling and inhibition of apoptosis. Further studies are needed in animal models of cancer to elucidate all possible protective mechanisms of hesperetin on the sorafenib antitumor activity. This may warrant clinical evaluation of hesperetin as a promising adjunct therapy in cancer patients receiving sorafenib.

## Supporting information

S1 Table Contains all data used in the study. (XLSX)

S1 Fig Contains all western blotting uncropped pictures. (PDF)

## List of abbreviations

NLR: Nod-like receptor
NOD: Nucleotide-binding oligomerization domain
TNF-α: tumor necrosis factor-α
IL-1β: interleukine 1-β
IL-6: interleukin 6
TLR4: Toll-like receptor 4
NF-kB: nuclear factor-kappa
NLRP3: nucleotide-binding oligomerization domain
LRR: leucine-rich repeat
