# Supplementary figures and images for: Hesperetin mitigates sorafenib-induced cardiotoxicity in mice through inhibition of the TLR4/NLRP3 signaling pathway
Source: PLoS One. 2022 Aug 9;17(8):e0271631. doi: 10.1371/journal.pone.0271631 (PMC9362940; doi:10.1371/journal.pone.0271631)

Beta actin

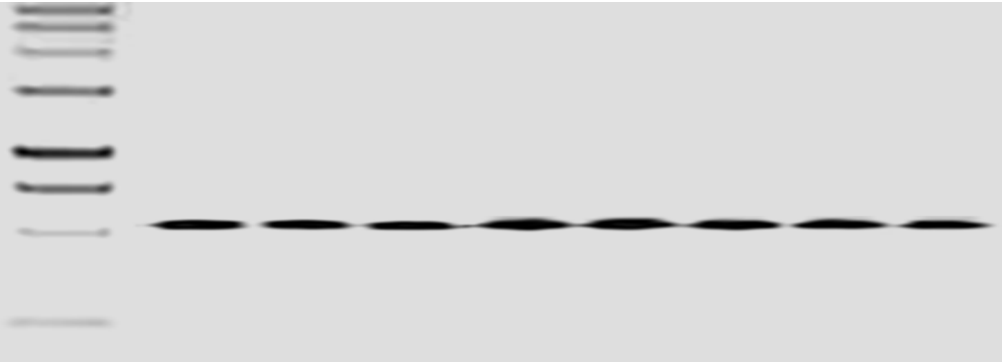

NLRP3

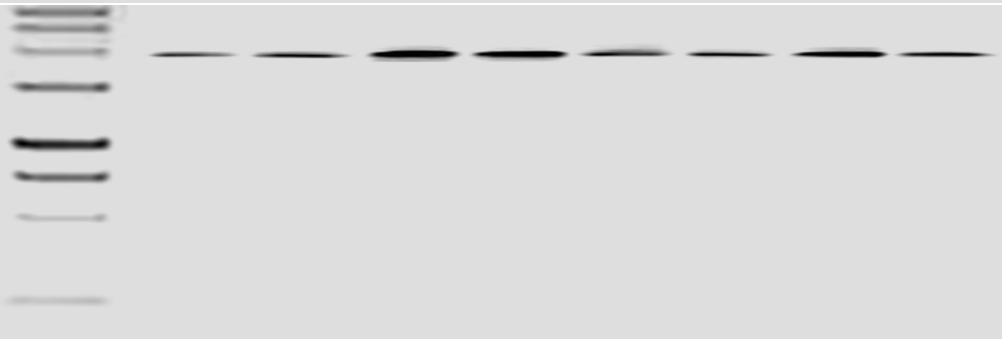

TLR4

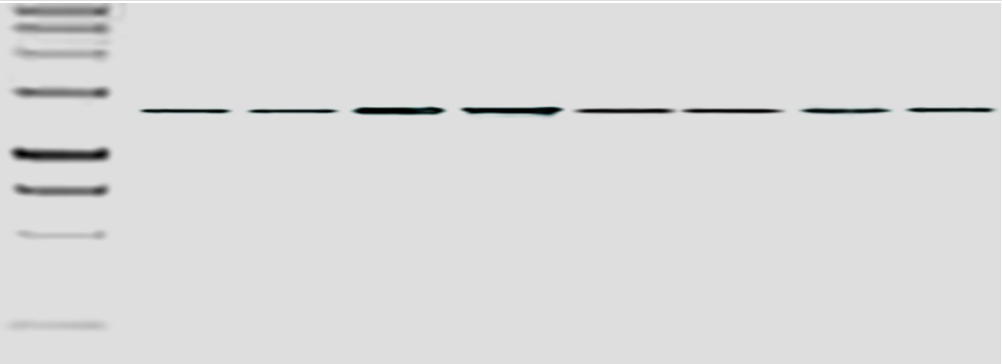

TGF-b

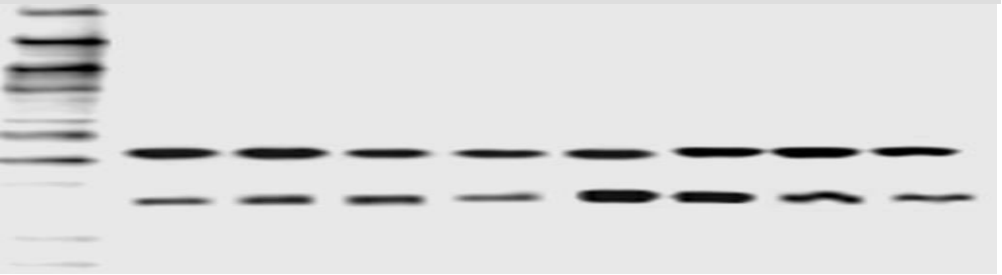

Caspase-3

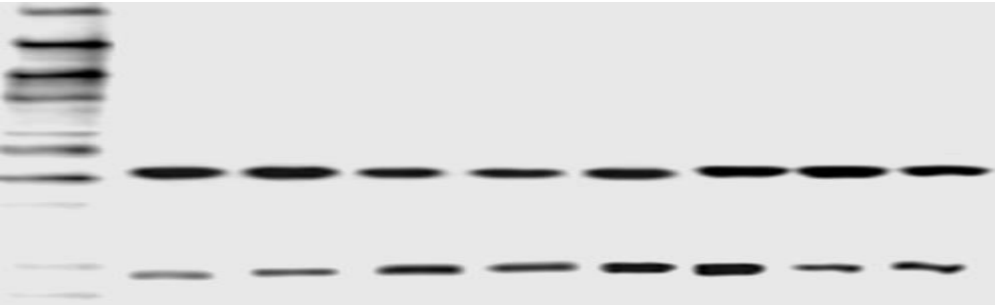

Bcl-2

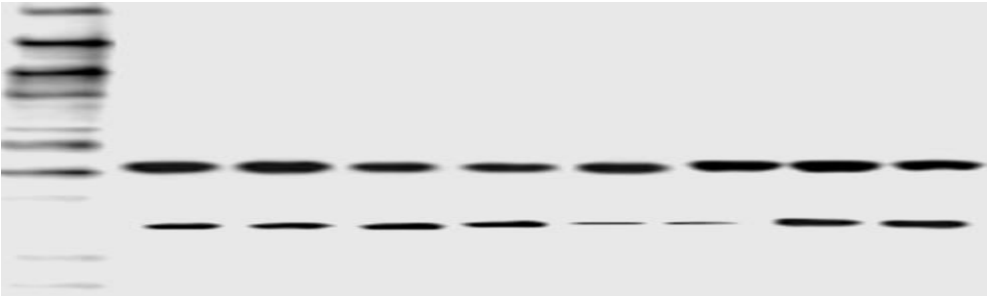

Supplement: S1 Fig — (PDF) [file pone.0271631.s002.pdf]
